# Prognostic Impact of Tumor-Infiltrating Lymphocytes, Tertiary Lymphoid Structures, and Neutrophil-to-Lymphocyte Ratio in Pulmonary Metastases from Uterine Leiomyosarcoma

**DOI:** 10.1245/s10434-023-14176-x

**Published:** 2023-09-01

**Authors:** Naoki Matsuda, Hiromasa Yamamoto, Tomohiro Habu, Kazuma Iwata, Kei Matsubara, Shin Tanaka, Kohei Hashimoto, Kazuhiko Shien, Ken Suzawa, Kentaroh Miyoshi, Tomohiro Toji, Mikio Okazaki, Seiichiro Sugimoto, Katsuhito Takahashi, Shinichi Toyooka

**Affiliations:** 1 Department of General Thoracic Surgery and Breast and Endocrinological Surgery, Okayama University Graduate School of Medicine, Dentistry and Pharmaceutical Sciences, Okayama, Japan; 2 Department of Thoracic Surgery, Okayama University Hospital, Okayama, Japan; 3 Organ Transplant Center, Okayama University Hospital, Okayama, Japan; 4 Center for Innovative Clinical Medicine, Okayama University Hospital, Okayama, Japan; 5 Department of Pathology, Okayama University Graduate School of Medicine, Dentistry and Pharmaceutical Sciences, Okayama, Japan; 6 Department of Pathology, Okayama University Hospital, Okayama, Japan; 7 Department of Sarcoma Medicine, Center for Sarcoma Multidisciplinary Treatment, Kameda Medical Center, Kamogawa, Chiba Japan

## Abstract

**Background:**

The presence of tumor-infiltrating lymphocytes (TILs) and tertiary lymphoid structures (TLSs) in tumor tissue has been related to the prognosis in various malignancies. Meanwhile, neutrophil-to-lymphocyte ratio (NLR) as a systemic inflammation marker also has been associated with the prognosis in them. However, few reports have investigated the relationship between pulmonary metastases from sarcoma and these biomarkers.

**Methods:**

We retrospectively recruited 102 patients undergoing metastasectomy for pulmonary metastases from uterine leiomyosarcoma at Okayama University Hospital from January 2006 to December 2019. TILs and TLSs were evaluated by immunohistochemical staining of surgically resected specimens of pulmonary metastases using anti-CD3/CD8/CD103/Foxp3/CD20 antibodies. NLR was calculated from the blood examination immediately before the most recent pulmonary metastasectomy. We elucidated the relationship between the prognosis and these factors. Because we considered that the status of tumor tissue and systemic inflammation were equally valuable, we also assessed the impact of the combination of TILs or TLSs and NLR on the prognosis.

**Results:**

As for TILs, CD3-positive cells and CD8-positive cells were correlated with the prognosis. The prognosis was significantly better in patients with CD3-high group, CD8-high group, TLSs-high group, and NLR-low group, respectively. The prognosis of CD8-high/NLR-low group and TLSs-high/NLR-low group was significantly better than that of CD8-low/NLR-high group and TLSs-low/NLR-high group, respectively.

**Conclusions:**

CD3-positive TILs, CD8-positive TILs, TLSs, and NLR are correlated with the prognosis, respectively. The combination of CD8-positive TILs or TLSs and NLR may be the indicators to predict the prognosis of patients with pulmonary metastases from uterine leiomyosarcoma.

**Supplementary Information:**

The online version contains supplementary material available at 10.1245/s10434-023-14176-x.

Uterine sarcomas are rare tumors, accounting for 3–7% of uterine malignancies and are less than 1% of all malignancies from female genital organs.^1^ Uterine leiomyosarcomas account for approximately 70% of all uterine sarcomas.^2^ After surgical resection, more than 90% of patients with pathological stage II, III, and IV will recur or progress.^3^ The majority of recurrences are in the abdomen and the pelvis, but metastases to the lungs also are common.^2^ Generally, a systemic treatment, such as chemotherapy, is appropriate for patients with pulmonary metastases. However, effective chemotherapy or molecular-targeted drugs are yet to be developed. When recurrent lesions can be resected completely, surgical resection can be the best strategy.^4^ After the first pulmonary metastasectomy (PM), sarcomas often relapse in the lungs. It was reported that the repeat of metastasectomy could bring good prognosis.^5^

Immunotherapy has taken a center stage as a novel cancer treatment approach.^6^ Immunotherapy provides the unprecedented opportunity to effectively treat, and even cure, several previously untreatable malignancies.^7^ An anticancer, immune response enables cancer cells to be killed by a series of stepwise events, including trafficking of T cells to tumors, infiltration of T cells into tumors, and recognition of cancer cells by T cells.^8^ The introduction of immune-checkpoint inhibitors for the treatment of numerous different types of cancer has created enormous interest among cancer immunologists and oncologists.^9^ In recent years, the researches of antitumor immunity have achieved a remarkable development. For an anticancer, immune response to lead to effective killing of cancer cells, a series of stepwise events mentioned above must be initiated and allowed to proceed and expand iteratively.^8^ Cytotoxic T cells, natural killer cells, B cells, and dendritic cells play important roles in these steps.^8,10,11^ Tumor-infiltrating lymphocytes (TILs) are thought to be an important indicator, reflecting the local immune-related tumor microenvironment (TME).^12^ Many studies have reported that TILs are associated with a good prognosis.^13–16^

Tertiary lymphoid structures (TLSs) are ectopic lymphoid organs that can develop at sites of chronic inflammation, such as those associated with infection and autoimmunity, but also form within the TME.^17^ TLSs share similar structural and functional characteristics with secondary lymphoid organs. TLSs are usually located in the invasive margin or in the stroma rather than the tumor core. TLSs can be identified as CD20-postive, B-cell follicles by immunohistochemistry.^18^ The prognostic impact of TLSs has been widely explored. Most reports have indicated that TLSs are associated with positive immunoreactivity and favorable clinical outcomes in most types of malignancies.^19–21^

Conversely, as the tumor grows, nonspecific, inflammatory responses caused by malignant cells or surrounding tissue tend to increase the peripheral blood neutrophil count and reduce the lymphocyte count.^22^ Systemic inflammatory responses, including the high neutrophil-to-lymphocyte ratio (NLR), are related to tumor development and progression and have been shown to be associated with outcomes in patients with various malignancies.^22^ We previously reported the NLR immediately before the most recent PM could be a novel independent prognostic factor for patients who underwent surgical resection for pulmonary metastases from various sarcomas.^23^

The purpose of this study was to investigate whether TILs, TLSs, and NLR were correlated with the prognosis of patients with pulmonary metastases from uterine leiomyosarcoma.

## Patients and Methods

### Patient Selection

This retrospective study was approved by Ethics Committee, Okayama University Graduate School of Medicine, Dentistry and Pharmaceutical Sciences and Okayama University Hospital, Okayama, Japan (approval number: K1612-033). Oral consent was given wherever possible from the study patients regarding the analyses using the surgically resected specimens, as there is no invasiveness for specimen collection. For deceased patients, we posted information-disclosing documents with the opportunity for the patients or family members of the deceased to opt out of this study on the website of Ethics Committee. Regarding the patients for whom we had obtained consent of the secondary use of specimen for the comprehensive gene analyses targeting various malignancies (approval number: K1603-066 and K1906-033), we also posted information-disclosing documents with the opportunity to reject this study. We maintain a database of the patients undergoing PM for pulmonary metastases from various primary sarcomas in Okayama University Hospital. Using our updated database, we conducted a retrospective review of a total of 256 patients who underwent PM between January 2006 and December 2019. Of the 256, 103 patients were diagnosed with uterine leiomyosarcoma as primary sarcoma. As specimens of the most recent PM for one patient did not remain in Okayama University Hospital, this patient was excluded. Thus, the final cohort consisted of 102 patients.

Patients were diagnosed with uterine leiomyosarcoma by histological examination of the primary lesion, and the presence of pulmonary metastasis was confirmed by histological examination of the surgical samples from PM. Patients who underwent PM for pulmonary metastases from uterine leiomyosarcoma met the following criteria: (a) the primary tumor was completely resected; (b) all metastatic diseases were completely resectable or controllable with local therapies; (c) the patients had a suitable performance status; (d) the planned procedure entailed acceptable anticipated complications; and (e) the respiratory function of the patients was sufficient to tolerate planned pulmonary resection.

### Data Collection

Clinical data for each patient were collected for the following variables: age at the first PM, histology; the date of the first PM; the maximum number of resected tumors among all the surgical procedures performed; the size of the largest lesion resected, frequency of pulmonary metastasectomy that each patient underwent as of the end of 2019; disease-free interval (DFI) from the date of the initial surgery for primary disease until the date of diagnosis of the first pulmonary metastasis; NLR immediately before the most recent PM; TILs and TLSs in the surgical specimens from the most recent PM. We evaluated TILs and TLSs by using the specimen of the biggest tumor in case of multiple lesions. The overall survival (OS) was calculated as the time interval from the first PM until death or the last recorded follow-up. All the patients were observed either until death or December 31, 2022.

### Immunohistochemical Staining of Surgically Resected Specimens

We defined the clusters of CD20-positive B cells located inside the tumor, near the margin of the tumor, and in the stroma of the tumor as TLSs. Tumor specimens in paraffin-embedded tissue were cut into 4-µm-thick sections. After the sections were deparaffinized by xylene, they were hydrated by a series of decreasing concentrations of ethanol. The antigens were activated by citrate buffer (Antigen Unmasking Solution, Vector, Newark, CA). Endogenous peroxidase activity was blocked by incubation in a 3% H_2_O_2_ solution. After ultrapure water washing, nonspecific reaction was blocked using blocking serum (Normal Horse Serum Blocking Solution, Vector). The sections were reacted with a rabbit monoclonal anti-CD3 antibody (clone: D7A6E™;1/200; CST, Danvers, MA), a mouse monoclonal anti-CD8 antibody (clone: C8/144B; 1/200; CST), a rabbit monoclonal anti-CD103 antibody (clone: EP206; 1/200; CST), a mouse monoclonal anti-Foxp3 antibody (clone: 236A/E7; 1/200; Abcam, Cambridge, UK), or a rabbit monoclonal anti-CD20 antibody (clone: E7B7T; 1/200; CST) at 4 °C overnight. The sections were incubated with a secondary antibody (ImmPRESS HRP Horse Anti-Rabbit or Anti-Mouse IgG Polymer Detection Kit, Peroxidase, Vector) for 30 min at room temperature. After washing in phosphate-buffered saline, the sections were visualized using 3-3′-diamino-benzidine (DAB Peroxidase Substrate Kit, ImmPACT, Vector) for 40 s and counterstained with hematoxylin.

### Evaluation of Immunohistochemical Staining

Tumor sections stained with anti-CD3, anti-CD8, anti-CD103, and anti-Foxp3 antibodies were scanned at ×20 magnification. Five fields of view were randomly selected. We counted the number of positive cells and calculated the average number of CD3, CD8, CD103, and Foxp3-positive cells in five fields. Tumor sections stained with an anti-CD20 antibody were scanned at ×10 magnification to select three fields with the greatest area of intratumoral and peritumoral CD20-positive cells and the percentage area (%) of each field with CD20-positive cells was calculated by using ImageJ software (Version 1.53, National Institutes of Health, Bethesda, MD). We defined the median as the cutoff value in all these five markers.

### Statistical Analyses

All statistical analyses were performed with EZR version 1.54 (Saitama Medical Center, Jichi Medical University, Saitama, Japan), a graphical user interface for R version 4.0.3 (The R Foundation for Statistical Computing, Vienna, Austria).^24^ Specifically, the software is a modified version of R commander (version 2.7-1), designed to add statistical functions frequently used in biostatistics.

Kaplan-Meier methods were used for calculating OS from the first PM. In terms of TILs and TLSs, we defined the median as the cutoff value. The cutoff value of NLR was determined by using a time-dependent receiver operating characteristic (ROC) curve analysis. A value of *p *< 0.05 was considered statistically significant. In case of multigroup comparison, we performed Bonferroni’s multiple comparison test.

## Results

### Patients’ Characteristics

We reviewed the medical record of 102 patients with pulmonary metastases from uterine leiomyosarcoma who underwent PM. Table 1 shows their clinical characteristics. Median age at the first PM was 52 (range 29–80) years. The mean and median NLR values immediately before the most recent PM were 2.32 and 2.01 (range 0.70–10.68), respectively.

**Table 1:** Characteristics of the patients undergoing pulmonary metasestasectomy (PM) for pulmonary metastases from uterine leiomyosarcoma (*n *= 102)

Characteristics	Results
Age at the first PM	
Median (range)	52 (29–80)
Neutrophil-to-lymphocyte ratio (NLR) immediately before the most recent PM	
Mean/Median (range)	2.32/2.01 (0.70–10.68)
Frequency of PM in each patient (2006–2019)	
1	58 (56.9%)
2	27 (26.4%)
3	12 (11.8%)
4 or more	5 (4.9%)
Maximum number of resected tumors	
Median (range)	3 (1–37)
≤ 5	69 (67.6%)
≥ 6	33 (32.4%)
Size of the largest lesion resected (mm)	
Mean/median (range)	16/27.2 (5–110)
≤ 10	23 (22.5%)
10–20	36 (35.3%)
> 20	43 (42.2%)
Disease-free interval	
Mean/median (range) (days)	548/296 (0–3653)
≤ 2 years	80 (78.4%)
> 2 years	22 (21.6%)

### TILs and TLSs

**Fig. 1:**
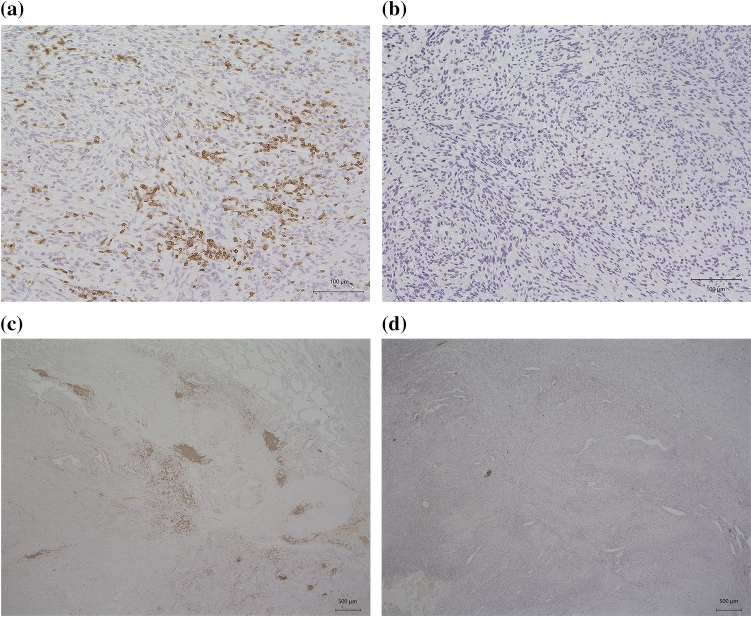
Representative images of CD3-positive tumor-infiltrating lymphocytes (TILs) and tertiary lymphoid structures (TLSs) evaluated by immunohistochemical staining. **a** CD3-high TILs. **b** CD3-low TILs. **c** TLSs-high specimen. **d** TLSs-low specimen

**Fig. 2:**
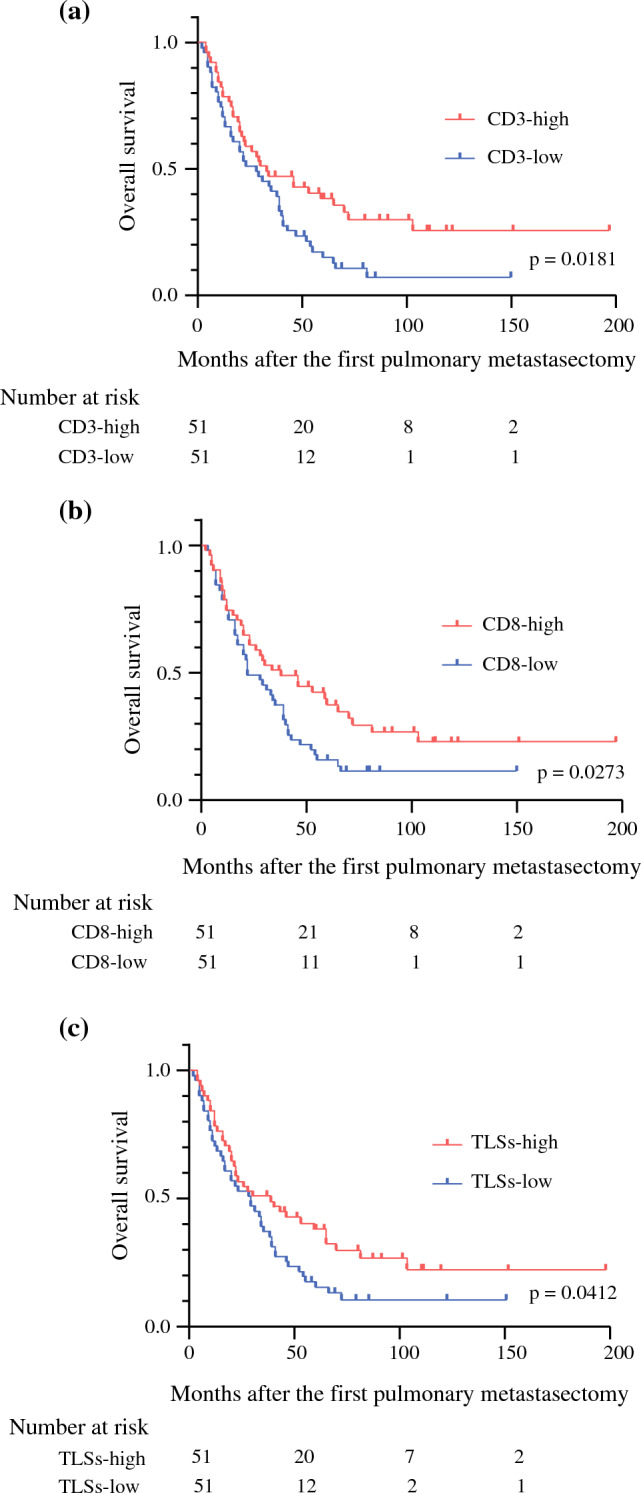
**a** Overall survival (OS) stratified by the status of CD3-positive tumor-infiltrating lymphocytes (TILs). **b** Overall survival (OS) stratified by the status of CD8-positive tumor-infiltrating lymphocytes (TILs). **c** Overall survival (OS) stratified by the status of tertiary lymphoid structures (TLSs)

We performed immunohistochemical staining of surgically resected specimens to evaluate TILs and TLSs. Representative images are shown in Fig. 1(a: CD3-high, b: CD3-low, c: TLSs-high, d: TLSs-low). We performed the ROC curve analysis to determine the cutoff value for TILs and TLSs. However, the area under the curve (AUC) was small; thus, we did not select the ROC curve analysis to decide the cutoff value. Instead, we defined the median as the cutoff value in all TILs and TLSs markers. The cutoff value of CD3-positive TILs, CD8-positive TILs, CD103-positive TILs, and Foxp3-positive TILs were 42.5, 31.7, 3.2, and 2.9 per field, respectively. The cutoff value of TLSs (CD20-positive density) was 0.367% per field. We divided the patients into two groups according to the cutoff value: CD3-high group (*n *= 51) and CD3-low group (*n *= 51); CD8-high group (*n *= 51) and CD8-low group (*n *= 51); CD103-high group (*n *= 52) and CD103-low group (*n *= 50); Foxp3-high group (*n *= 51) and Foxp3-low group (*n *= 51); and TLSs-high group (*n *= 51) and TLSs-low group (*n *= 51). The 5-year OS was significantly better in the patients with CD3-high group (38.1%) than in the patients with CD3-low group (15.0%) (*p *= 0.0181) (Fig. 2a). The 5-year OS was significantly better in the patients with CD8-high group (37.4%) than in the patients with CD8-low group (15.7%) (*p *= 0.0173) (Fig. 2b). The 5-year OS was significantly better in the patients with TLSs-high group (38.0%) than in the patients with TLSs-low group (15.4%) (*p *= 0.0412) (Fig. 2c). In terms of the OS for CD103 and Foxp3, there was no significant difference between the two groups (Supplementary Figs. 1a-b).

### NLR

**Fig. 3:**
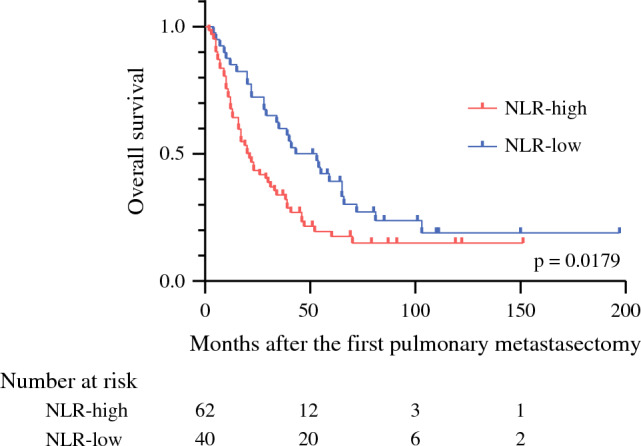
Overall survival (OS) stratified by the status of neutrophil-to-lymphocyte ratio (NLR)

A time-dependent ROC curve was introduced at 3 years from the first PM to determine the optimal cutoff value for NLR immediately before the most recent PM. The cutoff value was defined as the point on the curve that was closest to the upper left corner of the plot. The optimal cutoff value for NLR was 1.88 (area under the curve [AUC] = 0.619, 95% confidence interval [CI] 0.509–0.729) (Supplementary Fig. 2). We divided the patients into two groups according to the cutoff value: NLR-high group (*n* = 62) and NLR-low group (*n* = 40). The 5-year OS was significantly better in the patients with NLR-low group (39.3%) than in the patients with NLR-high group (17.7%) (*p* = 0.0179) (Fig. 3).

### Combination of CD8-positive TILs or TLSs and NLR

**Fig. 4:**
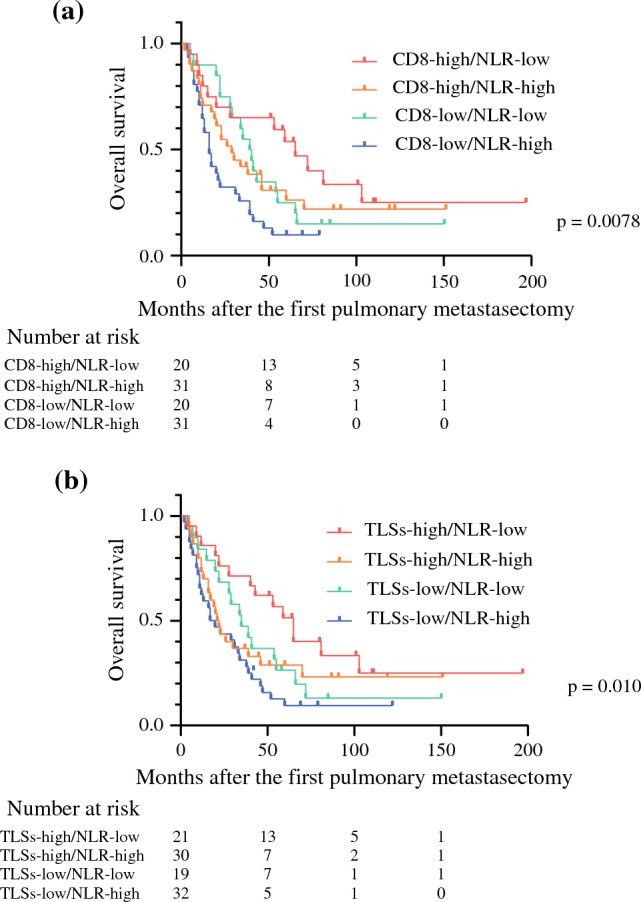
**a** Overall survival (OS) stratified by the combination of CD8-positive tumor-infiltrating lymphocytes (TILs) and neutrophil-to-lymphocyte ratio (NLR). **b** Overall survival (OS) stratified by the combination of tertiary lymphoid structures (TLSs) and neutrophil-to-lymphocyte ratio (NLR)

We found that CD3-positive TILs, CD8-positive TILs, TLSs, and NLR were correlated with the survival of patients undergoing PM for pulmonary metastases from uterine leiomyosarcoma. We performed an interaction analysis of CD8-positive TILs or TLSs and NLR. However, there was no interaction between them (CD8-positive TILs and NLR; *p* = 0.702, TLSs and NLR; *p* = 0.512) (Supplementary Figs. 3a, b), indicating that the status of tumor tissue and systemic inflammation were independent. Therefore, we assessed the impact of the combination of CD8-positive TILs or TLSs and NLR on the prognosis of patients undergoing PM for pulmonary metastases from uterine leiomyosarcoma. We divided the patients into four groups according to the combination of these factors, respectively. Regarding CD8-positive TILs and NLR, the OS of CD8-high/NLR-low group was significantly better than that of CD8-low/NLR-high group (*p* = 0.0078) (Fig. 4a). The 5-year OS was 53.6% in CD8-high/NLR-low group. This result was better than that of only CD8-high group (37.4%) or that of only NLR-low group (39.3%). In terms of TLSs and NLR, the OS of TLSs-high/NLR-low group was significantly better than that of TLSs-low/NLR-high group (*p* = 0.010) (Fig. 4b). The 5-year OS was 51.6% in TLSs-high/NLR-low group. This result was better than that of only TLSs-high group (38.0%) or that of only NLR-low group (39.3%).

## Discussion

In this study, we assessed TME and systemic inflammation status of patients undergoing PM for pulmonary metastases from uterine leiomyosarcoma by means of tumor tissue and a peripheral blood count. Our results suggest that TILs, TLSs, and NLR are good predictors of the prognosis in patients undergoing PM for pulmonary metastases from uterine leiomyosarcoma. However, it is not sufficient to extract the group of the patients with better prognosis by using only one factor. Therefore, we introduced some combinations of biomarkers from tumor tissue and peripheral blood to predict survival in patients undergoing PM for pulmonary metastases from uterine leiomyosarcoma more accurately.

We explored whether TILs were correlated with the prognosis using some markers that distinguish the subtype of TILs. Although it was reported all the markers we used in this study could be prognostic factors in several malignancies respectively, only CD3 and CD8 were correlated with the prognosis in our study. The immune microenvironment is composed primarily of TILs, macrophages, and dendritic cells.^25^ All components play critical roles in the detection and elimination of tumor cells, thereby contributing to arresting tumor progression and proliferation. The activation of T cells requires the T-cell receptor complex, which includes the co-receptor CD3.^26^ The CD3-positive cells are likely reflecting immunocompetent T lymphocytes with antitumor activity; thus, CD3-positive TILs can be a prognostic factor. CD8-positive T cells play a pivotal role in the adaptive immune response to malignancy.^14^ Activated CD8-positive T cells are named cytotoxic T lymphocytes (CTL) as they can directly recognize and kill malignant and infected cells.^27^ The primary antitumor activity of CTL is to kill target cells via the exocytosis of cytotoxic granules containing perforin and granzymes as well as the production of cytokines such as interferon-γ and tumor necrosis factor.^28^ Thus, CD8-positive TILs also can be a prognostic factor.

In this study, we show that the presence of more TLSs can be a better prognostic factor. TLSs are crucial components of TME. They facilitate the recruitment of immune cells. For example, TLSs around a tumor play a crucial role in inducing antitumor, cytotoxic, T-cell proliferation,^29^ acting as a predictor for the prognosis of various malignancies.^29,30^ There are many reports about the density of TLSs on the primary lesion, such as breast cancer, gastric cancer, and endometrial cancer.^31–33^ Although there are limited data on the presence and function of TLSs at metastatic sites, several reports describe the characteristics of TLSs in metastatic sites. TLSs in metastases seem to depend on both the primary tumor type and the organ in which the metastatic site is located. In the study of breast cancer that analyzed a large cohort of 355 metastases from four metastatic sites (lung, liver, brain, and ovary), TLSs were found in lung and liver metastases but not in brain and ovarian metastases.^34^ TLS densities in pulmonary metastases from different tumor types are highly variable and seem to mirror those of the primary sites, being at high densities in metastases from colon and prostate cancers, intermediate densities in metastases from thyroid, renal, liver, and breast cancers, and low densities in metastases from leiomyosarcoma and osteosarcoma.^35^ It may be significant to identify the group with better prognosis by extracting the cases with TLSs-high in the worse-prognosis disease, such as uterine leiomyosarcoma.

We found low NLR immediately before the most recent PM could be a good prognostic factor in uterine leiomyosarcoma. The NLR is a systemic inflammation marker, which has been reported to be an independent prognostic factor for survival in several malignancies.^22^ The impact of neutrophils and lymphocytes on the tumor microenvironment is increasingly recognized. Previous studies have shown that large numbers of neutrophils can inhibit the activation and cytolytic antitumor activity of lymphocytes and natural-killer cells.^36^ For tumor patients, the NLR reflects not only the systemic inflammation but also the immune state to tumors.

Although there are several studies that demonstrated TILs or TLSs were correlated with the preoperative NLR,^31,37^ there was no relationship between TILs or TLSs and NLR in this study. However, even in the studies that showed the correlation between TILs or TLSs and NLR, they did not suggest the reasonable mechanism, warranting further study. Instead, we consider that TME and the status of systemic inflammation are independent factors that affect the prognosis.

In this study, we combined TILs or TLSs and NLR to evaluate prognosis. Then, we were able to extract patients with better prognosis more accurately. Pulmonary metastases from uterine leiomyosarcoma often exist bilaterally, and they frequently recur in the lung after PM. Thus, several cases need repeat PM. In this cohort, 40% of all the patients underwent plural PM. These combined factors may be effective as not only prognostic factors but also indicators to decide surgical indication.

Although the role of immune checkpoint inhibitors in sarcoma treatment is unclear, some studies have demonstrated the efficacy of immune checkpoint inhibitors to sarcoma.^38,39^ Meanwhile, there is a study that the presence of TLSs predicted the efficacy of immune checkpoint inhibitor in solid tumors.^40^ If immune checkpoint inhibitors become a treatment option to uterine leiomyosarcoma, evaluating TLSs and TILs may be effective to predict responsiveness to the drugs.

To fully explore the role of TILs and TLSs to malignancies, it may be better to evaluate not only those of metastatic lesions but also those of primary lesions. However, there is a report that TLSs at metastatic sites have the same characteristics as in primary sites. Thus, regarding the prognostic impact of TLSs, those in metastatic sites parallel that of primary tumors.^35^ Therefore, evaluating only TLSs of pulmonary metastases seems to be beneficial.

There are some limitations in this study. Regarding the evaluation of TLSs, we simply explored the CD20-positive cells, although there are several methods to evaluate the details of TLSs, such as CD21-positive cells and CD23-positive cells that classify the phenotype of TLSs, several T-cell markers contained in TLSs, and the chemokines associated with TLSs.^13,19,20^ If we evaluate TLSs in detail as mentioned above, we may elucidate the function of TLSs on pulmonary metastases from uterine leiomyosarcoma. Although this is currently the biggest cohort of patients with pulmonary metastases from uterine leiomyosarcoma in which TILs and TLSs are assessed, it might still be underpowered. A multi-institutional study with a considerable sample size will be necessary to further clarify the prognostic value of TILs and TLSs in pulmonary metastases from uterine leiomyosarcoma.

## Conclusions

We identified that the high CD3-positive TILs, the high CD8-positive TILs, the high density of TLSs, and the low NLR immediately before the most recent PM were better prognostic factors, respectively. Moreover, the combination of these TILs or TLSs and NLR resulted in clarifying the groups with better prognosis. Further investigations are warranted to clarify tumor microenvironment in pulmonary metastases from uterine leiomyosarcoma.

### Supplementary Information

Below is the link to the electronic supplementary material.

Supplementary Fig. 1a Overall survival (OS) stratified by the status of CD103-positive tumor-infiltrating lymphocytes (TILs) (TIF 7699 KB)

Supplementary Fig. 1b Overall survival (OS) stratified by the status of Foxp3-positive tumor-infiltrating lymphocytes (TILs) (TIF 7693 KB)

Supplementary Fig. 2 Receiver operating characteristic (ROC) curve analysis to determine the cutoff value of neutrophil-to-lymphocyte ratio (NLR). AUC area under the curve (TIF 7605 KB)

Supplementary Fig. 3a Linear-regression analysis for CD8-positive tumor-infiltrating lymphocytes (TILs) and neutrophil-to-lymphocyte ratio (NLR) (TIF 7595 KB)

Supplementary Fig. 3b Linear-regression analysis for tertiary lymphoid structures (TLSs) and neutrophil-to-lymphocyte ratio (NLR) (TIF 7582 KB)
