# Supplementary figures and images for: Prognostic Impact of Tumor-Infiltrating Lymphocytes, Tertiary Lymphoid Structures, and Neutrophil-to-Lymphocyte Ratio in Pulmonary Metastases from Uterine Leiomyosarcoma
Source: Ann Surg Oncol. 2023 Sep 1;30(13):8727–34. doi: 10.1245/s10434-023-14176-x (PMC10625945; doi:10.1245/s10434-023-14176-x)

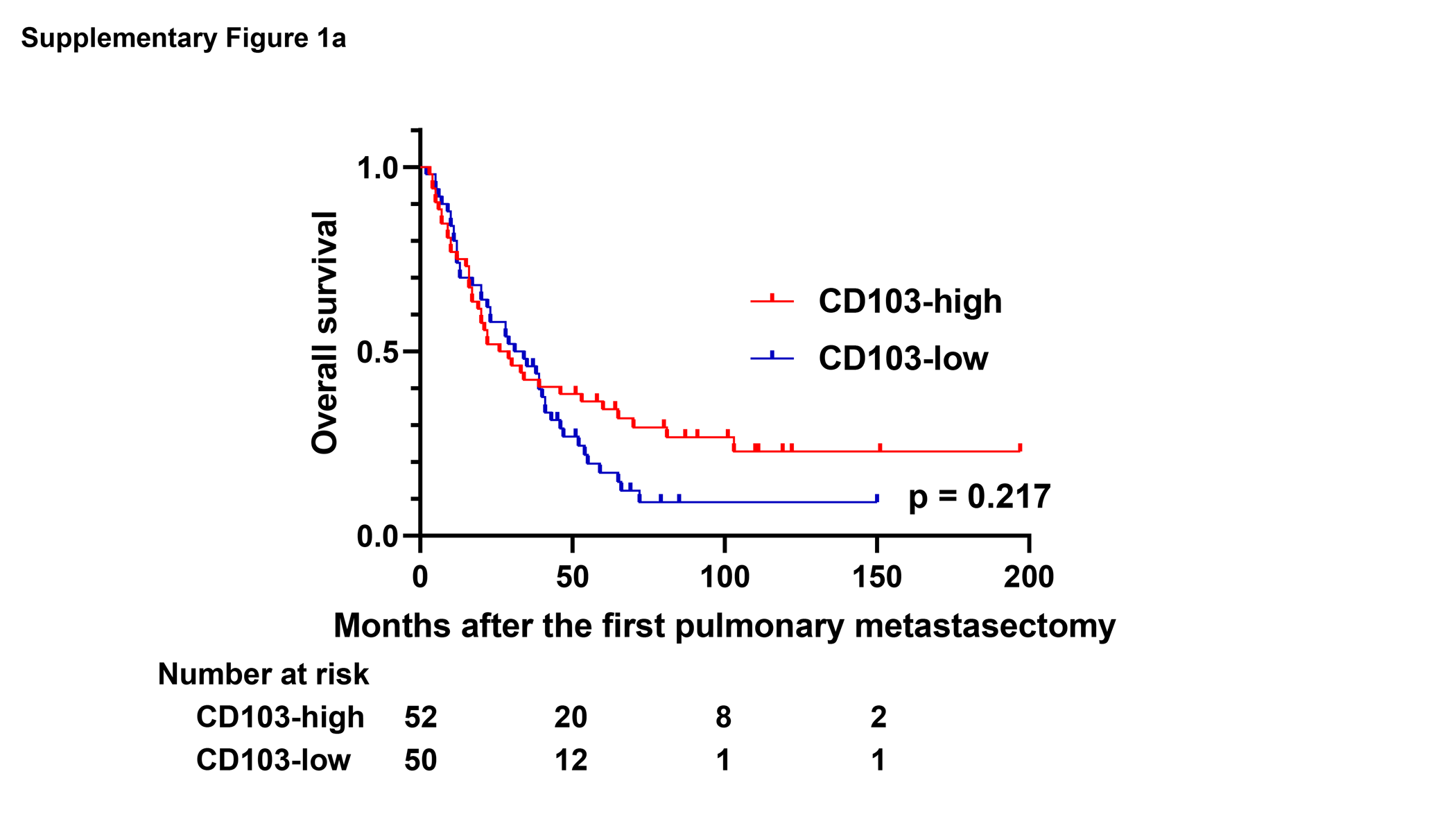

Supplement: Supplementary file 1 — Supplementary Fig. 1a Overall survival (OS) stratified by the status of CD103-positive tumor-infiltrating lymphocytes (TILs) (TIF 7699 KB) [file 10434_2023_14176_MOESM1_ESM.tif]

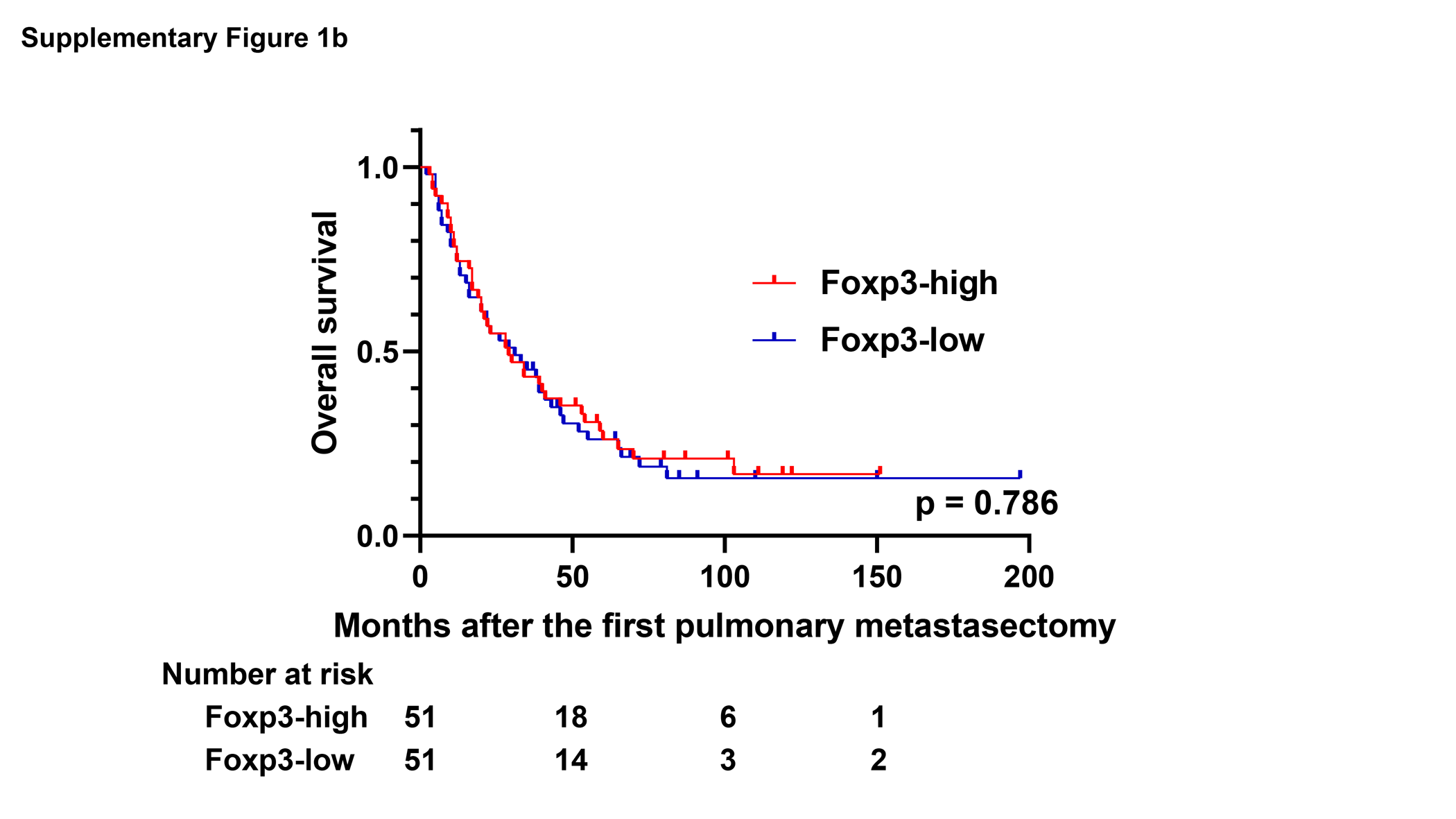

Supplement: Supplementary file 2 — Supplementary Fig. 1b Overall survival (OS) stratified by the status of Foxp3-positive tumor-infiltrating lymphocytes (TILs) (TIF 7693 KB) [file 10434_2023_14176_MOESM2_ESM.tif]

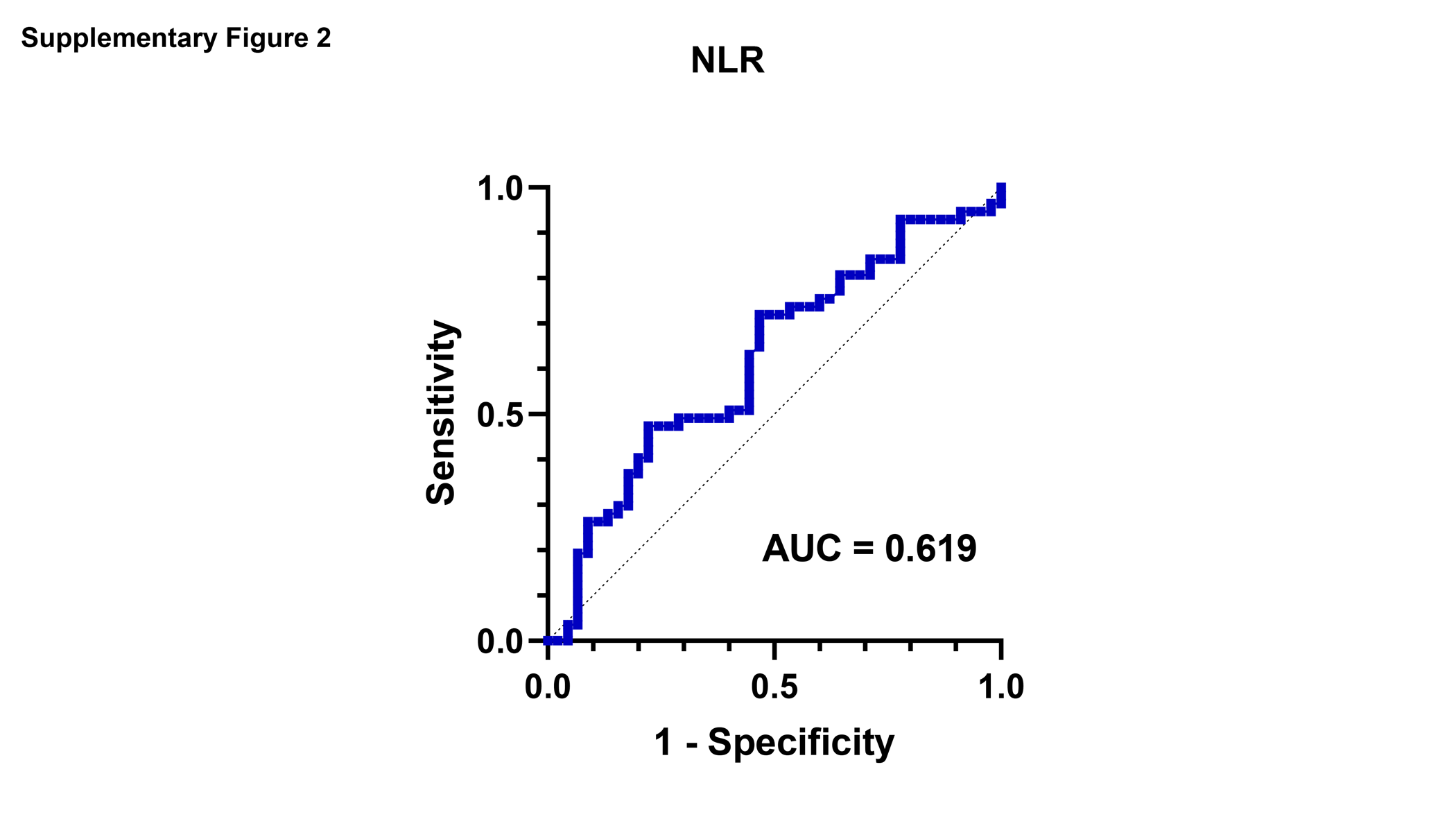

Supplement: Supplementary file 3 — Supplementary Fig. 2 Receiver operating characteristic (ROC) curve analysis to determine the cutoff value of neutrophil-to-lymphocyte ratio (NLR). AUC area under the curve (TIF 7605 KB) [file 10434_2023_14176_MOESM3_ESM.tif]

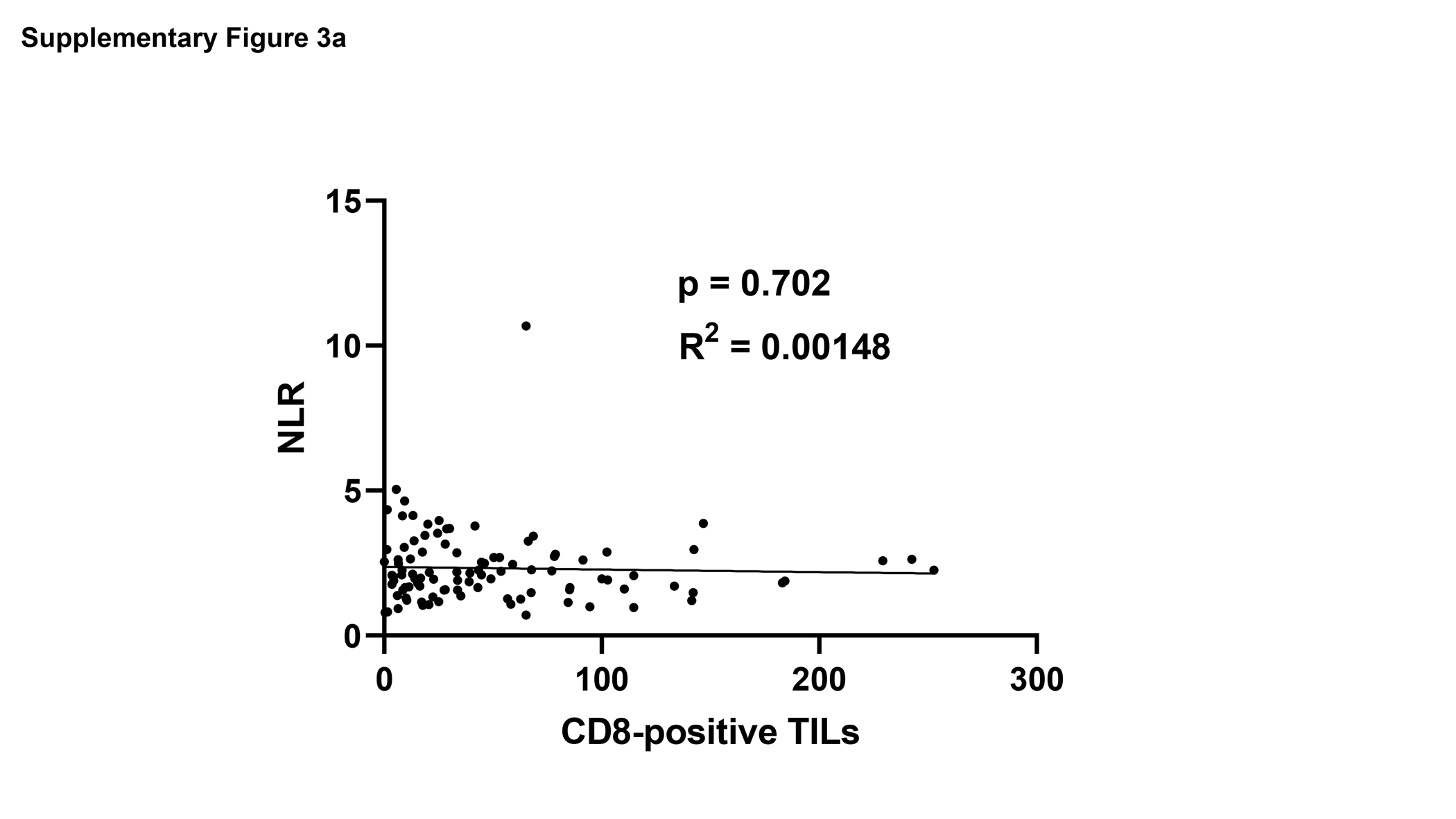

Supplement: Supplementary file 4 — Supplementary Fig. 3a Linear-regression analysis for CD8-positive tumor-infiltrating lymphocytes (TILs) and neutrophil-to-lymphocyte ratio (NLR) (TIF 7595 KB) [file 10434_2023_14176_MOESM4_ESM.tif]

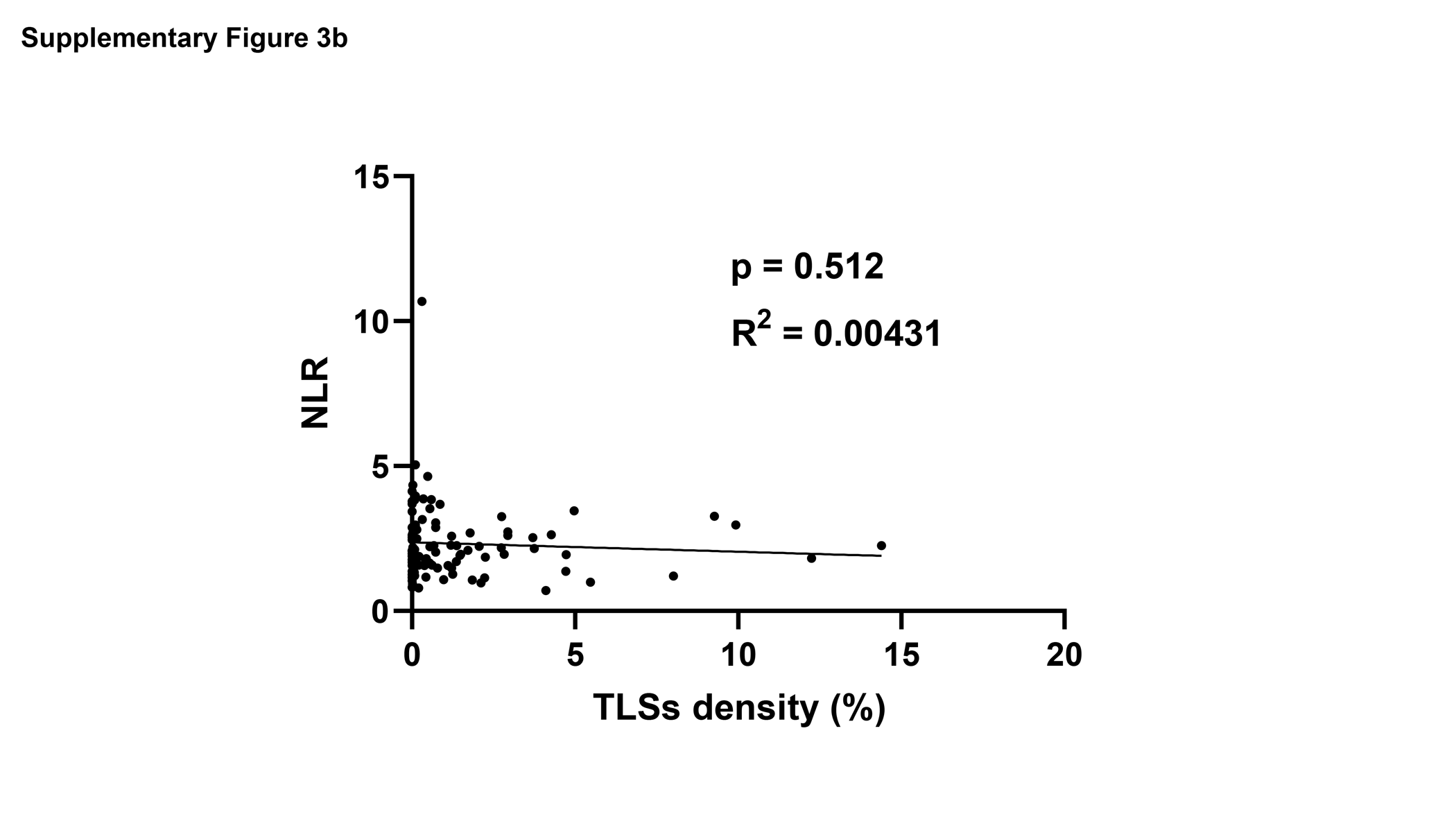

Supplement: Supplementary file 5 — Supplementary Fig. 3b Linear-regression analysis for tertiary lymphoid structures (TLSs) and neutrophil-to-lymphocyte ratio (NLR) (TIF 7582 KB) [file 10434_2023_14176_MOESM5_ESM.tif]
